# Skin Cancer: Epidemiology, Screening and Clinical Features of Acral Lentiginous Melanoma (ALM), Melanoma *In Situ* (MIS), Nodular Melanoma (NM) and Superficial Spreading Melanoma (SSM)

**DOI:** 10.7150/jca.116362

**Published:** 2025-09-21

**Authors:** Chun-Te Lu, Teng-Li Lin, Arvind Mukundan, Riya Karmakar, Anusha Chandrasekar, Wen-Yen Chang, Hsiang-Chen Wang

**Affiliations:** 1 Institute of Medicine, School of Medicine, College of Medicine, National Yang Ming Chiao Tung University, No. 155, Sec. 2, Li-Nong Street, Beitou District, Taipei 112304, Taiwan.; 2 Department of Surgery, Division of Plastic and Reconstructive Surgery, Taichung Veterans General Hospital, 1650 Taiwan Boulevard Sect. 4, Taichung 407219, Taiwan.; 3 Department of Dermatology, Dalin Tzu Chi General Hospital, No. 2, Min-Sheng Rd., Dalin Town, Chiayi 62247, Taiwan.; 4 Department of Mechanical Engineering, National Chung Cheng University, 168, University Rd., Min Hsiung, Chia Yi 62102, Taiwan.; 5 Department of Biotechnology, Karpagam Academy of Higher Education, Salem - Kochi Hwy, Eachanari, Coimbatore, Tamil Nadu 641021, India.; 6 Department of General Surgery, Kaohsiung Armed Forces General Hospital, 2, Zhongzheng 1st.Rd., Lingya District, Kaohsiung City 80284, Taiwan.; 7 Department of Medical Research, Dalin Tzu Chi Hospital, Buddhist Tzu Chi Medical Foundation, No. 2, Minsheng Road, Dalin, Chiayi 62247, Taiwan.; 8 Director of Technology Development, Hitspectra Intelligent Technology Co., Ltd., Kaohsiung 80661, Taiwan.

**Keywords:** Skin Cancer, Acral Lentiginous Melanoma, Melanoma *in situ*, Nodular Melanoma, and Superficial Spreading Melanoma, Machine Learning, Artificial Intelligence

## Abstract

Melanoma, a highly aggressive form of skin cancer, presents considerable challenges in early detection and accurate diagnosis, particularly across its diverse subtypes such as acral lentiginous melanoma (ALM), melanoma *in situ* (MIS), nodular melanoma (NM), and superficial spreading melanoma (SSM). This study assesses the epidemiology, clinical characteristics, and screening techniques related to various melanoma subtypes, emphasizing their distinct features and risk factors. Moreover, the use of machine learning (ML) methodologies to categorize melanoma subtypes and the thorough examination of advancements in AI-based melanoma diagnosis, primarily emphasizing convolutional neural networks (CNN) and transfer learning approaches. Evaluate the efficacy of several deep learning models in classifying melanoma subtypes while addressing significant obstacles, including class imbalance and model generalization. Furthermore, it contemplates the integration of multimodal data, including genetic information and patient demographics, to enhance diagnostic accuracy. This comprehensive review assesses the epidemiology, clinical characteristics, and machine learning techniques utilized for the classification and detection of different melanoma subtypes, emphasizing recent advancements in AI-driven methods and their clinical significance.

## Introduction

In humans, skin cancer is the most common cancer, with millions of cases diagnosed annually, particularly in the white population 1. Ultraviolet radiation (UVR) and radiotherapy or immunosuppressive therapy caused by environmental exposure, that results in skin cancer 2. The white populations are caused in 90 - 95% by UV radiation in skin cancer and therefore the population-attributable factors are considered to be predominantly 3. Generally, skin cancer is classified into melanoma and non-melanoma skin cancer (NMSC) will be developed by the derived cell 4. Melanoma highly deadly type of skin cancer because it causes most deaths and minority populations, melanoma occurs more commonly in unusual anatomic locations compared to white populations 5, 6. Compared to melanomas, non-melanomas are the largest common kind of skin cancer 7. Melanoma is a dangerous type of skin cancer, with a global death rate of 14%, and to the World Health Organization, nearly 7,650 deaths from melanoma were anticipated in 2022, and 99,780 new melanoma cases in the USA 8, 9. There are 1,958,310 newly diagnosed cancer cases and 609,820 cancer deaths in the United States in 2023 10. In the United States, 5.4 million new cases of skin cancer in every year 11. In 2020, the estimated 19.3 million new causes with a 95% uncertainty interval ranging between 19.0 to 19.6 million cancers and almost 10.0 million deaths from cancer, with a 95% uncertainty interval of 9.7 to 10.2 million 12.

Diagnosis of cutaneous cancer usually begins with a skin assessment, dermoscopy, patient history, and surgical biopsy 13. The management of skin cancer has a long and successful history in radiation therapy (RT), which is a complementary method in cutaneous oncology 14. The ABCBE mnemonic stands for asymmetry (A), border irregularity (B), color variability (C), diameter (D), and evolution (E) or any change. Additionally, the morphology, location of the body, and arrangement of lesions may also provide information about skin malignancy 15. There are four main types of skin melanoma: acral lentiginous melanoma (ALM), melanoma *in situ* (MIS), nodular melanoma (NM), and superficial spreading melanoma 16.

ALM is a subtype that is normally diagnosed at later stages due to poor attention to lesions arising on extremities and medical diagnostic mistakes 17. ALM occurs on acral skin, including the nail beds, soles, and palms 18. It is challenging to clinically diagnose ALM, particularly in its early stages, due to the subtle clinical and histopathologic changes 19. ALM subtypes are the most frequent types of malignant among Asian people and are found in people with dark skin tone, particularly in the soles of the feet 20. In individuals with Asian, black, or dark brown skin, the most common is ALM 21. ALM is rare in all ethnicities because other melanoma types are even less common in African Americans, Hispanics, and Asians, ALM represents a common melanoma seen in ethnic groups 22. ALM is the majority type of melanoma in several African, South American, and Asian countries it represents a moderately low percentage of melanoma cases in some countries. In populations of European descent, such as the United Kingdom, Australia, and the United States 23. MIS is a special challenge in histopathology, clinical management, and treatment 24. The incidence of MIS is increasing more rapidly than any invasive or *in situ* cancer in the US, and it represents the earliest form of melanoma where the malignancy is localized to the epidermis 25, 26. The prevalence of MIS is increasing as the population ages and risk factors, including immunosuppression and sun exposure, are becoming more common, making treatment increasingly necessary, as long-term cumulative sun exposure is linked to the development of MIS 27, 28. NM is the most popular type of melanoma, typically diagnosed between 40 and 50 years old, and similarly common in each sex. The trunk and neck are the most common locations for occurrences 29, 30. NM most commonly appears on the chest or back and tends to grow vertically in the skin, deeply penetrating if not removed 31. NM has a poor prognosis, and it includes 12% - 30% of all diagnosed melanomas, with the largest incidence rates in Australia and New Zealand 32, 33. SSM is the usual subtype of melanoma among fair-skin people, corresponding to 70% of cases, and it is a specific histologic subtype of cutaneous melanoma 34, 35. It begins an initial radial growth phase, characterized by a growth limited to the skin layer, then a depth growth phase that involves the presence of invasion 36. Between 1978 and 2007, the incidence and survival of SSM have increased, while the incidence and survival rates for NM have changed 37.

This research offers a comprehensive review that synthesizes existing knowledge about the utilization of machine learning and artificial intelligence approaches for the identification and classification of melanoma subtypes. This review critically evaluates existing methodologies, including deep learning models like convolutional neural networks and transfer learning approaches, to elucidate their merits, limitations, and practical issues, in contrast to original experimental findings. The report synthesizes recent research findings to present an overview of technical breakthroughs and emerging trends, while noting gaps and opportunities for further inquiry. This comprehensive analysis aims to aid physicians, researchers, and AI practitioners in comprehending the advancing domain of AI-enhanced melanoma diagnosis and to facilitate the creation of more precise and universally applicable diagnostic instruments.

## Clinical Features of ALM, MIS, NM, SSM

### Acral lentiginous melanoma (ALM)

**Figure 1:**
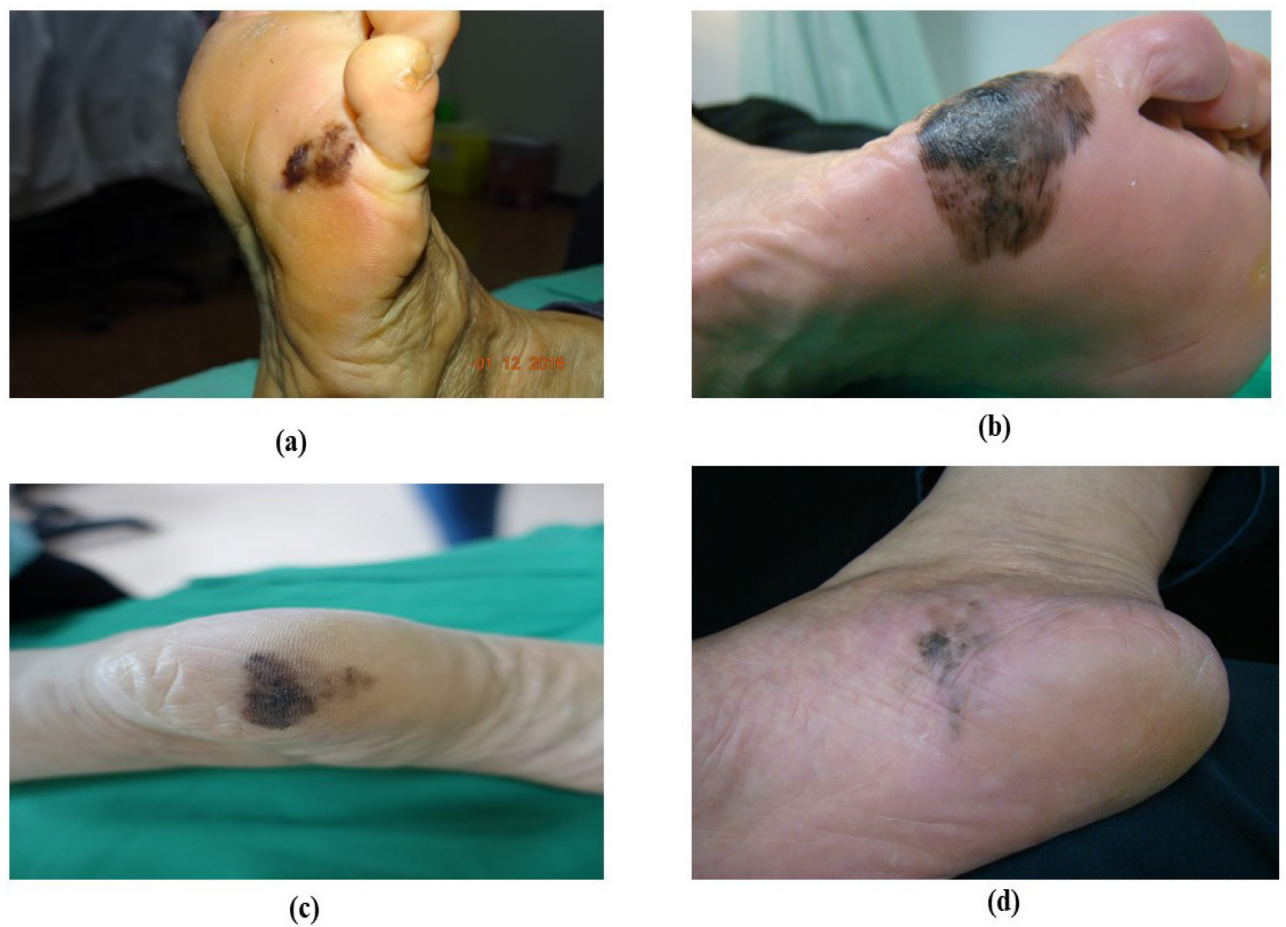
Acral lentiginous melanoma based on their appearance. (a) brownish ALM at the left sole, (b) blackish ALM at the right sole, (c) blackish ALM at the left sole (d) blackish ALM at the sole

**Figure 2:**
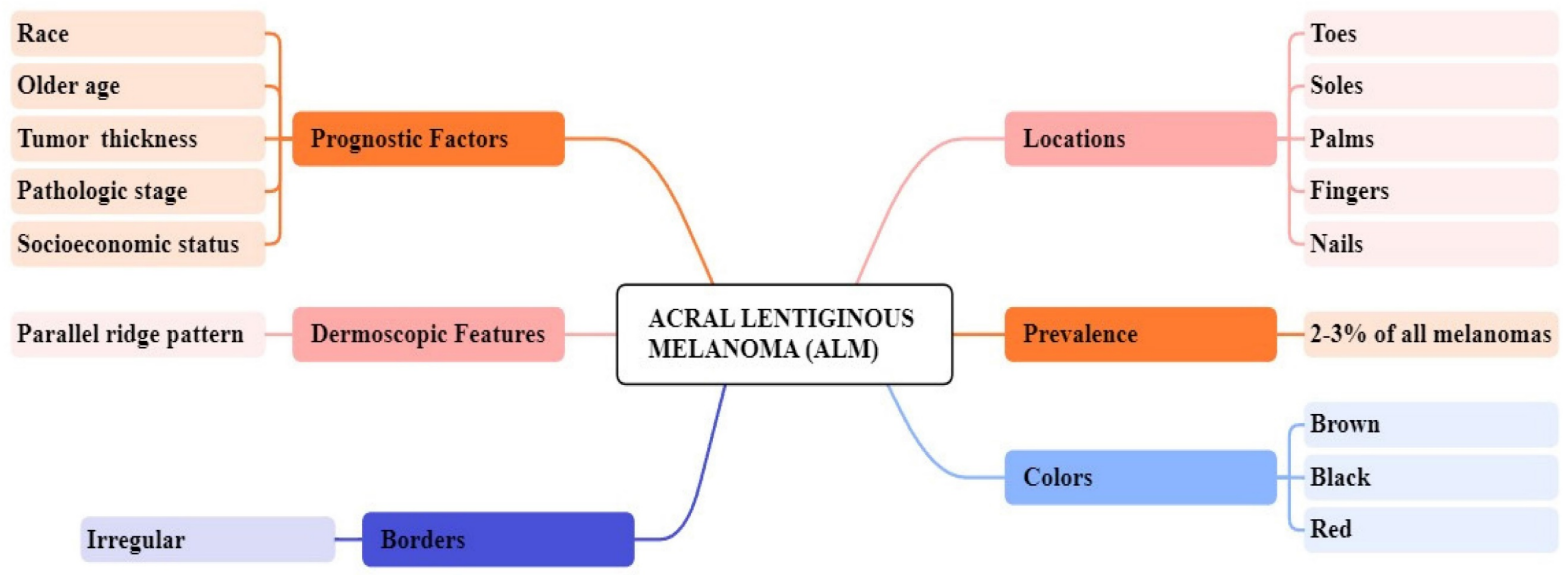
Overview of ALM clinical features

ALM represents about 2 to 3% of all melanoma cases and its uncommon form of melanoma is related to later diagnosis and lower survival percentages 38, 39. It is found beneath the nail plate or sole, toes, fingers, and hairless skin of palms. The colors that appear on ALM are brown, black, and red as shown in Figure 1 40. Clinically, ALM shows an initial radial growth phase, appearing as variegated pigmentation, and an uneven brown to-black macule 41. The unique dermatoscopic characteristic of ALM is the linear ridge pattern, which is characterized by linear pigmentation along the bands of volar skin as shown in Figure 2 42. Prognostic factors include older age, pathologic stage, tumor thickness, socioeconomic status, and race 43. Histopathologically, ALM is distinguished by lentiginous proliferation during the radial growth phase. Although rare, ALM represents around 10% of all melanomas 44. Subungual variants are rarer in the white-skinned population, with additional clinical features including clinical hypomelanosis, a family record of melanoma, hair color, and any previous history of non-melanoma skin cancer 45, 46.

### Melanoma *in situ* (MIS)

**Figure 3:**
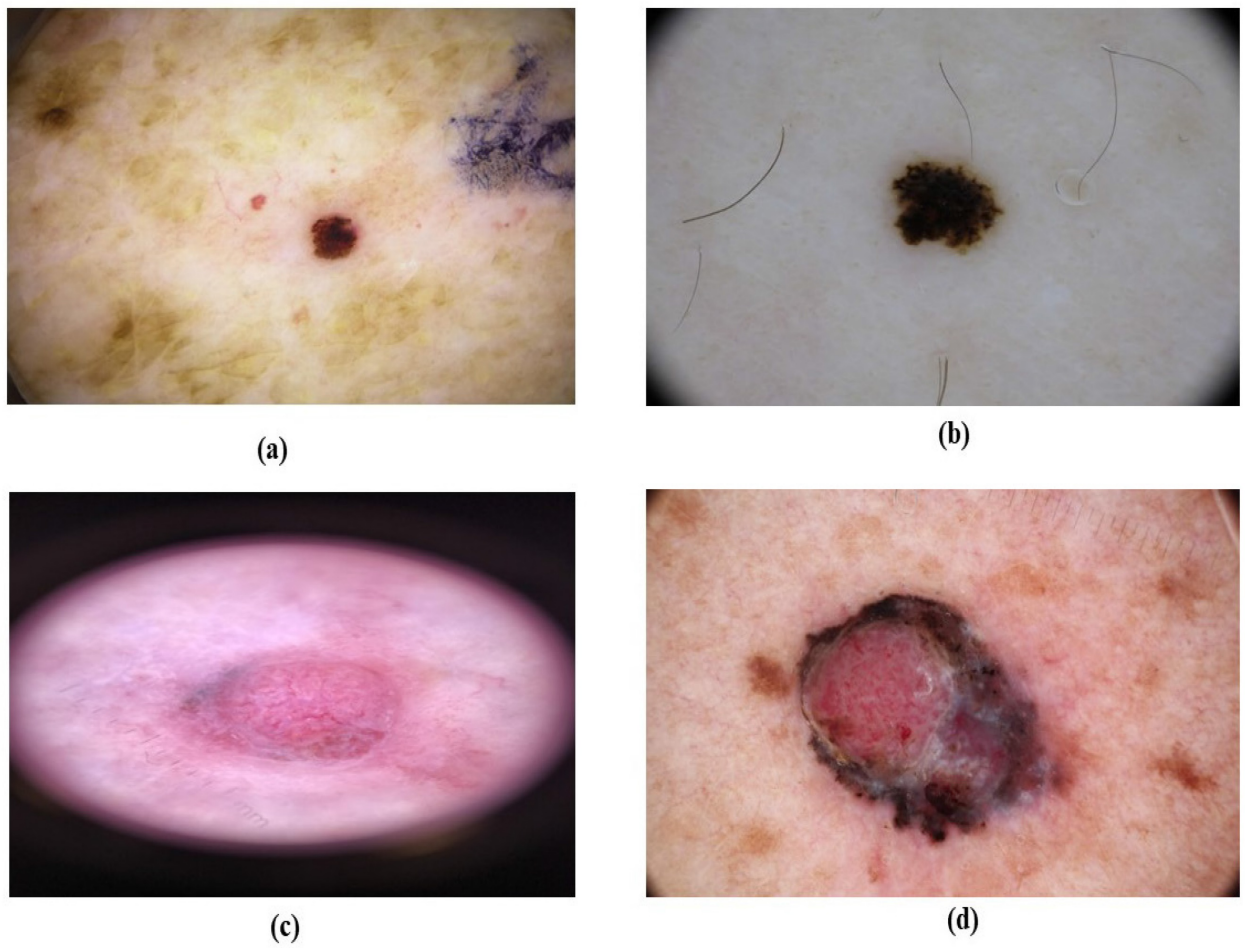
Melanoma *in situ* (MIS) based on their color. (a) brownish MIS (b) blackish MIS (c) pinkish MIS, (d) reddish MIS

**Figure 4:**
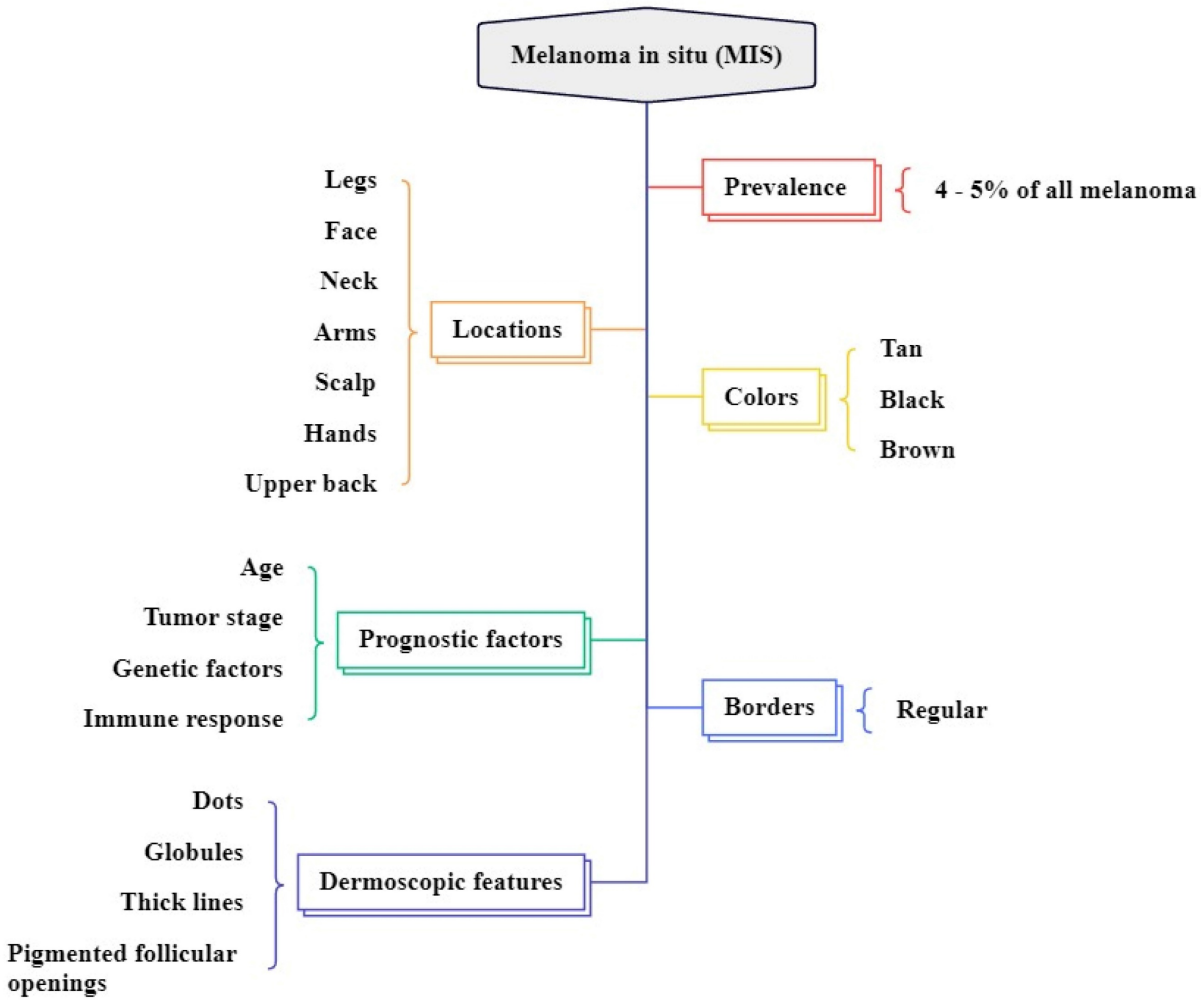
Overview of MIS clinical features

MIS accounts for 4% to 15% of all melanoma types and commonly arises in chronically sun-damaged areas of the skin, particularly in older individuals, and begins as a brown or tan macule as shown in Figure 3 47, 48. It is often found on the neck, face, and scalp of advanced-age patients with major sun-induced skin damage, and can also occur in non-head and neck regions like the legs, forearms, and back of the hands as shown in Figure 4 49, 50. The colors are variable shades of dark brown, tan, brown, and black 51. The dermatoscopic features are asymmetrically pigmented with follicular openings, dots, and globules aggregated around adnexal openings, thick pigmented lines, and an annular granular pattern 30. In difference to other standard types of MIS, the borders of lentigo maligna (LM) are regularly defined histologically and clinically, as the lesion usually combines with the surrounding area of long-term sun damage 52.

### Nodular melanoma (NM)

**Figure 5:**
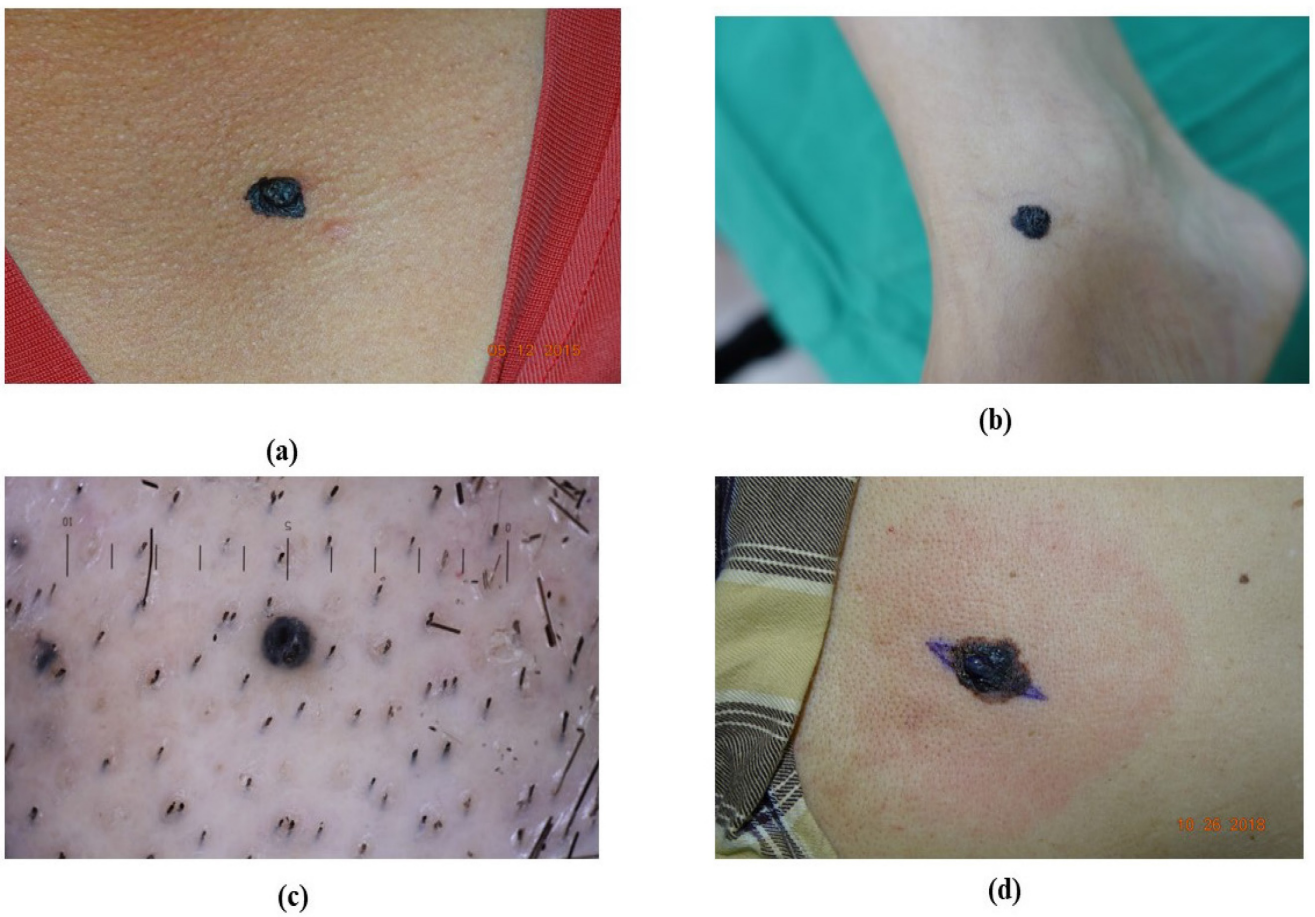
Nodular melanoma (NM) based on their location (a) NM at neck, (b) NM at leg, (c) NM at head, (d) NM at chest.

**Figure 6:**
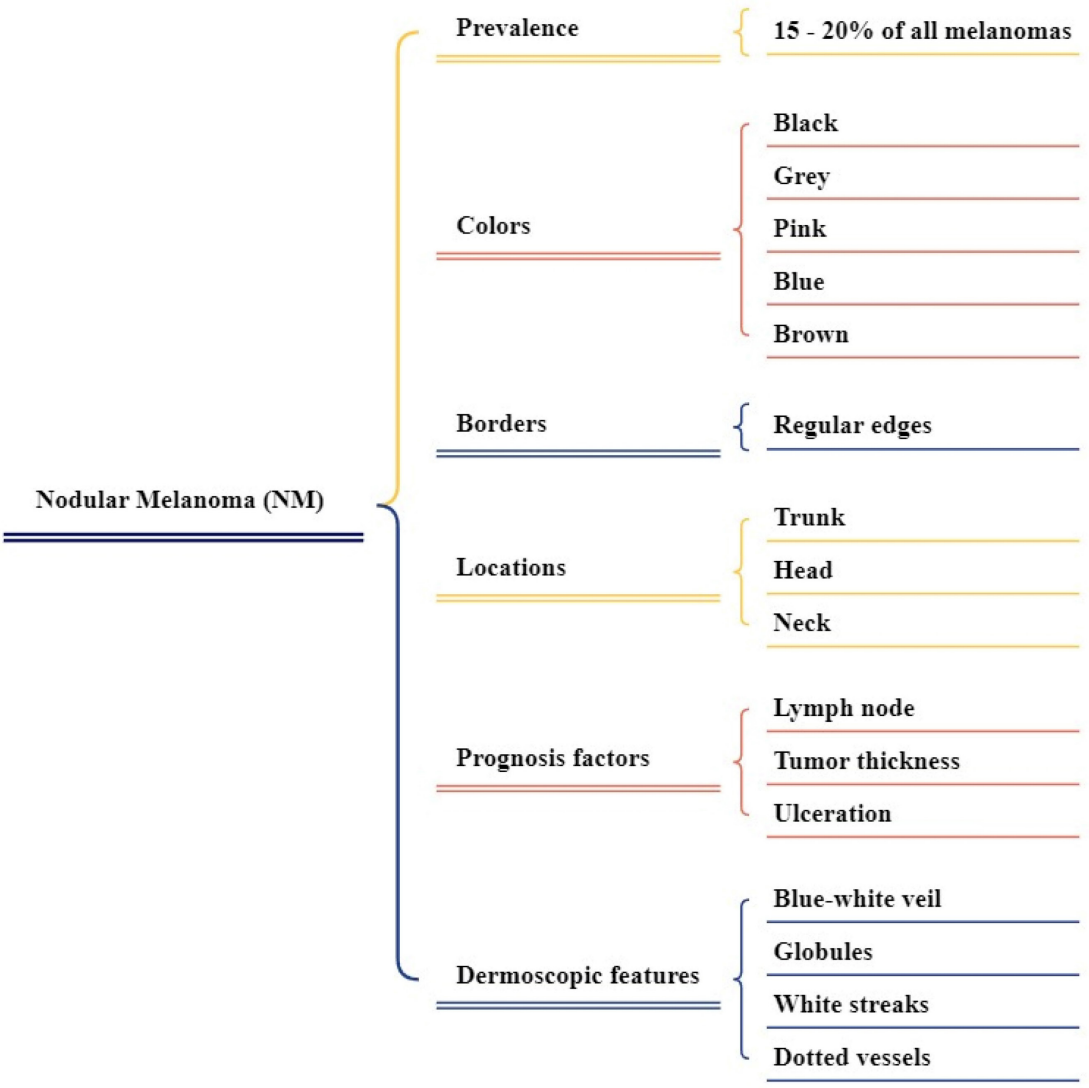
Overview of NM clinical features.

NM is the second greatest well-known type in light-skinned individuals, representing around 15 to 20% of melanoma cases. It is most generally located on the trunk, neck, or head, more common in males as shown in Figure 5 53. The colors found in NM vary from brown, blue, black, grey, and pink, including other shades of these color or their combinations. The surface of NM can be rough, scaly, or smooth as shown in Figure 6 54. Dermatoscopic features of NM include a blue-white veil, white streaks, isolated globules, and dotted vessels or uneven linear 55. NM indicates the lack of the early radial growth phase, which begins with perpendicular growth, and lesions are usually symmetric, with small diameters, regular borders, and uniform color 56, 57. In the Early stage, the injury is usually an irregular black or blue nodule with even edges 58. NM lesions are more common and lighter colored than other common melanoma subtypes 59.

### Superficial spreading melanoma (SSM)

**Figure 7:**
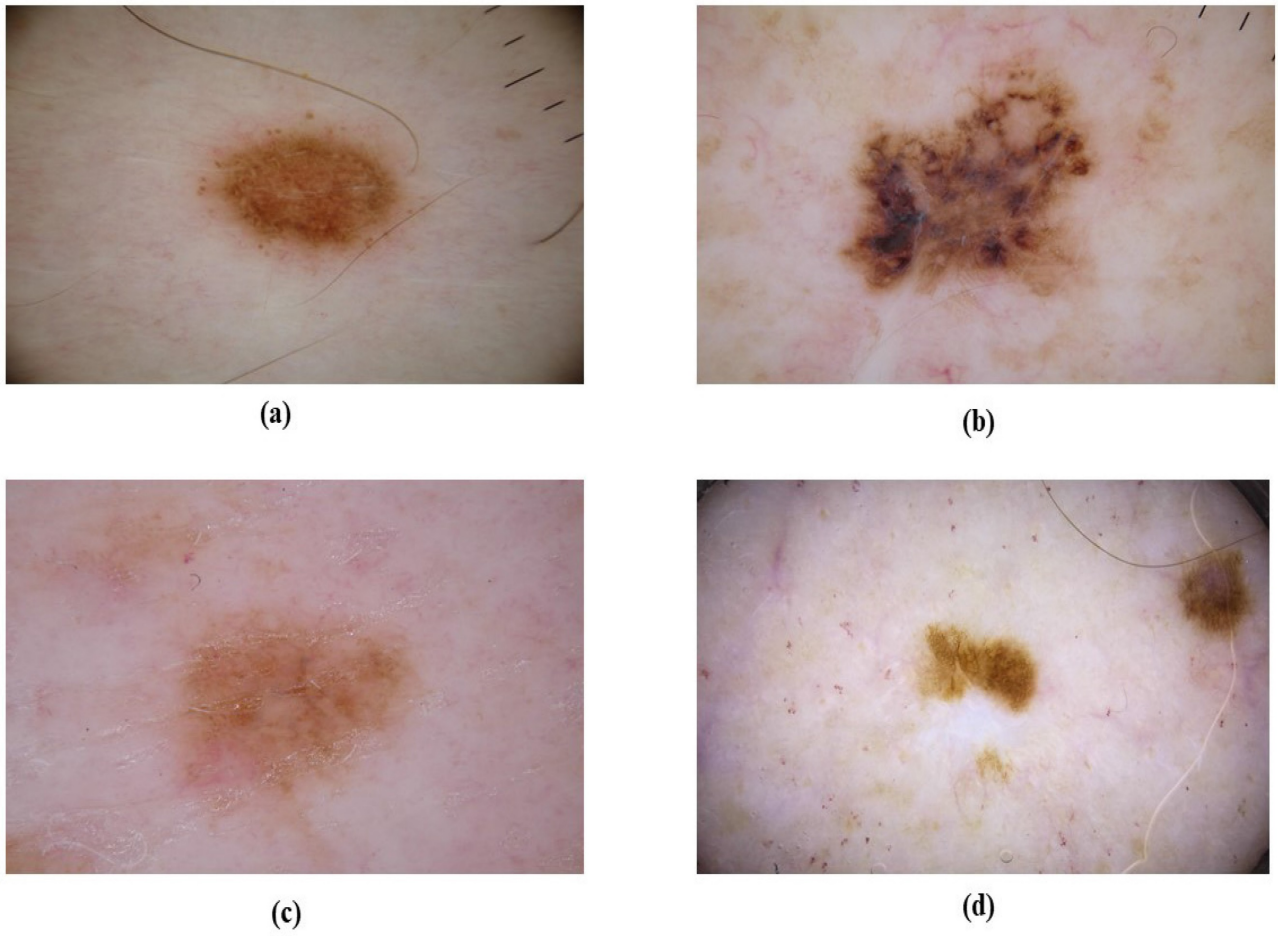
Superficial Spreading Melanoma (SSM) based on their shape(a) reddish SSM with round, (b) brownish SSM at irregular borders, (c) reddish SSM at irregular round, (d) brownish SSM at small irregular borders.

**Figure 8:**
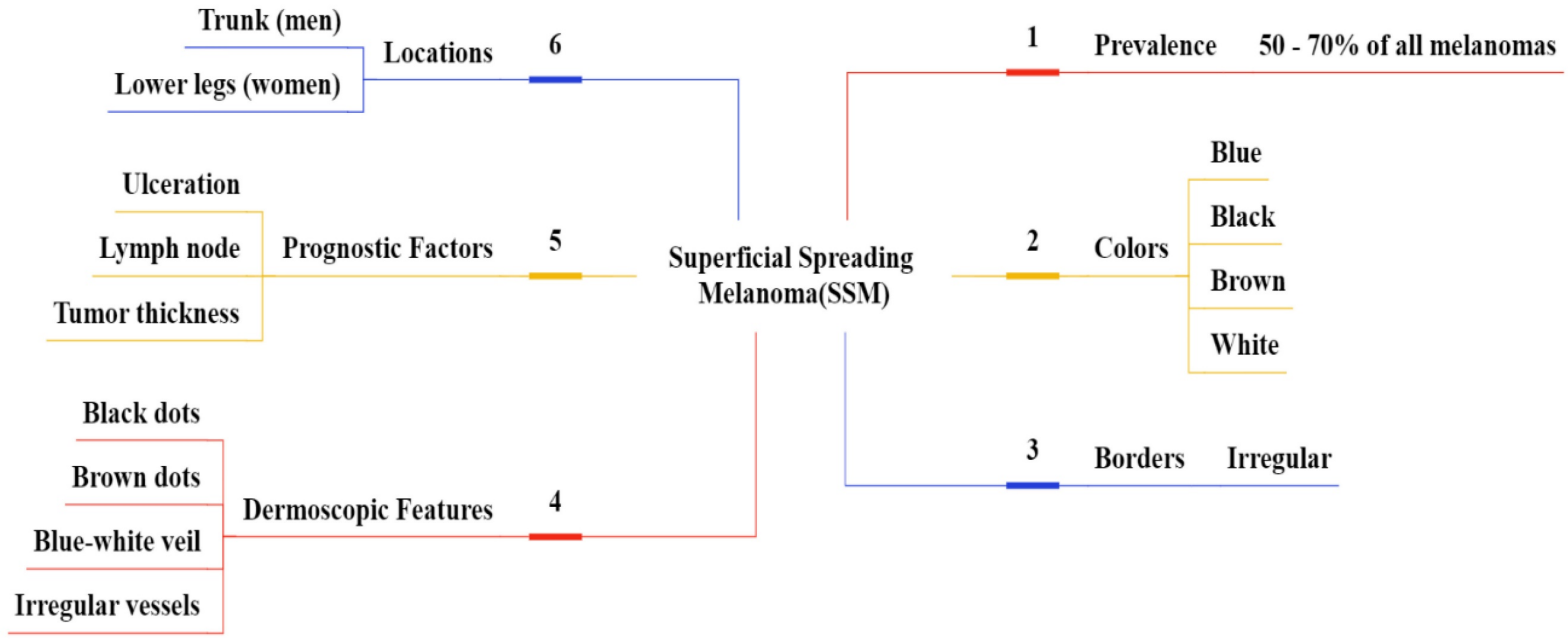
Overview of SSM clinical features.

SSM represents between 50% to 70% of melanoma cases and most commonly on the trunk in males and on the lower extremities in females 60, 61. SSM typically presents as a macule or plaque with an uneven border and variable pigmentation, its measures range from several millimeters to various centimeters, often displaying multiple colors like blue, red, gray, black, and white as shown in Figure 7 62. Dermatoscopic features are a blue-white layer, peripheral black dots, several brown spots, and irregular vascular structures as shown in Figure 8 63. Histopathological features of SSM include asymmetry, lack of cellular maturation, and poor circumscription 64.

## Screening and Diagnosis of ALM, MIS, NM, SSM

### Screening and Diagnosis of ALM

#### Etiology

The case of ALM remains uncertain, as familial cases have not been reported so far, there is diffused evidence indicating that some genetic risk factors may be present. For instance, a major longitudinal study observed that patients with degree relatives diagnosed with ALM had an increased risk of any major melanoma subtypes, indicating some shared genetic factors among various melanoma types 65. In some studies, dummy or shearing stress was suggested as a cause of the occurrence of the ALM this becomes clear because ALM is most commonly found in load-bearing zones of the foot, such as the heel, forefoot, and lateral side 66, 67. Ghanavatian et al., followed up on this and noted that adjusted for surface area, ALM occurrence was inversely proportional associated with atypical and benign acral nevi presence in these weight-bearing areas. The incidence of this phenomenon is much lower among men than women 68.

#### Epidemiology

ALM is in the range of 2 to 3% among newly identified melanomas, and the average age of diagnosis is 62.8 years. Changing ALM distribution seems to positively influence women's age, which generally begins when reaching 80 years old, and suddenly it increases within this age range. Male and female populations affected by ALM are reported to be approximately equal. However, females are reported relatively better than males in the diagnosis stage of ALM. Besides, among different subtypes of melanoma, ALM is much more prevalent among non-whites than other types 69. There appears to be a considerable difference in the frequency of ALM when compared to the other types of melanoma, across different ethnic-racial groups. Because of the shortage of available cases, the global patterns of ALM pancreatic cancer epidemiology have not been established, although it, in fact, usually, corresponds to the ethnicity of people residing within the territory. As an example, ALM comprises 55-58% of all the developmental melanoma types in Taiwan and Korea while in the United States, it is only about 2% 70. This difference, for the most part, concerns the whiter population who sustain higher rates of sun-derived melanomas. ALM is present in 1-8% of rare melanoma populations of European origin, and responsible for more than 50% of all cutaneous malignant melanoma cases 23.

#### Dermoscopy

The accurate clinical diagnosis of ALM has been greatly improved due to the dermoscopic analysis. Dermoscopic findings may be more crucial than histological images in the early stages of ALM 71. In contrast, classical dermoscopic patterns of acral melanocytic nevi include the parallel furrow pattern, lattice-like pattern, and fibrillar pattern, which are found in over 75% of benign acral lesions 72. While acquired acral nevi measure several millimeters in size, typically present symmetrically, and are flesh-colored, congenital acral nevi cover areas up to a few centimeters of the skin surface, often have an asymmetric pattern, and show blue-gray color or globules 73.

### Screening and diagnosis of MIS

#### Etiology

MIS is an early, non-invasive form of melanoma in which the tumor is uncertain to the epidermis 49. UVR exposure from sunlight and tanning beds is an important environmental risk since it increases the risk of melanoma 74. The development of melanoma is usually caused by genetic mutations in genes such as BRAF, NRAS, and others 75.

#### Epidemiology

MIS has increased the case strongly among white populations during the past few decades 76. The Surveillance, Epidemiology, and End Results (SEER) database indicates that MIS is one of the cancers that is developing very quickly, with an annual increase of 9.5% 77. In 2018, there will be nearly 2500 invasive and 1700 in-suit cases of melanoma in New Zealand, based on estimations 76. There are indications that many cases of melanoma are overdiagnosed, with overtreatment causing harm to the patients, apart from increasing medical costs for individuals and healthcare systems. Current estimates about the rate of overdiagnosis in Australia may be outdated, and the financial implications for the healthcare system have not yet been thoroughly examined 78. Over the past 40 years, the incidence of malignant melanoma has steadily increased, at a rate of approximately 5% a year. The current global incidence is reported at 10.9/100,000 persons with the lifetime risk of developing melanoma for Americans estimated to be 1 in 75. Though the overall survival rates specific to melanoma have improved over the last two decades, the prognosis for patients with advanced disease has not demonstrated any advancement compared to 20 years ago 79.

#### Pathophysiology

MIS is the initial stage of melanoma where the atypical melanocytes are confined within the epidermis and have not yet increased depth dermal layers 49. Genetic mutation, especially in the BRAF, NRAS, and CDKN2A genes, plays a very significant part in the development of melanoma. The genetic material coding for cyclin D1 (CCND1) plays a role through its interaction with normal cell cycle regulation 80. Among these mutations, the most important is undoubtedly the BRAF V600E mutation, which leads to the activation of the mitogen-activated protein kinase-MAPK pathway that basically pushes melanocyte proliferation and survival 81.

#### Dermoscopy

Recent literature suggests that dermatoscopy has increased the diagnostic precision of PSLs. The technique utilizes multiple criteria are specific patterns and structures both for melanocytic and non-melanocytic lesions, general asymmetry, and variability in color and structure 82. Schiffner et al., described four characteristic dermoscopic criteria as diagnostic patterns of LM pigment asymmetric follicular openings, dark rhomboidal structures, slate-colored globules, and slate dots. These characteristics combined achieve a sensitivity of CI of 89% and a specificity of 96% 82.

### Screening and diagnosis of NM

#### Etiology

The major risk factors include multiple dysplastic nevi, family history, fair skin that tends to burn, and sun exposure some studies also indicate that UV radiation is an important component of melanoma risk 83, 84. NM has been associated with genetic factors, specifically mutations in the CDKN2A and CDK4 genes, but it also reflects the impact of such environmental factors as sun exposure and dysplastic nevi 85.

#### Epidemiological

Epidemiological studies have shown that fast-growing NM is predominately found in men over the age of 50 presenting without the conventional risk factors for other types of cutaneous melanoma (CM), such as multiple nevi, freckles, or sun damage. Unfortunately, this group with high risk is largely not included in skin cancer screening programs. Moreover, these screening measures seem to have little impact on the early recognition of NM most tumors develop quickly *de novo* in other areas of the skin and are generally self-detected 86. Over the past few decades, invasive melanoma incidence has increased progressively in the white populations of the USA, United Kingdom, Australia, and New Zealand. Incidence rates are projected to continue to rise through 2031 in the majority of these populations. In contrast, over the same time, melanoma mortality has also been rising, but at a rate far lower than the incidence increase 87.

#### Pathophysiology

Melanoma has its origin in melanocytes at the dermal-epidermal junction where they undergo a malignant transformation. Although this cancer can arise from a pre-existing nevus, it often arises *de novo*. In summary, melanoma evolution is generally divided into two phases: the radial and vertical growth phases. The radial growth phase displays a horizontal alignment of neoplastic melanocytes at the intraepidermal plane which could extend to the papillary dermis as well 88. The vertical growth phase is characterized by dermal invasion and nodule formation of a tumor 89.

#### Dermoscopy

NM is challenging for dermoscopy because the pattern asymmetry is less significant than in SSM. However, pigmented NM usually found an uneven color 90. Argenziano et al., found a novel indicator of NM characterized by the blue-black color within the lesion. It is proposed that the blue-black color is due to a blend of pigments in the mid-deep dermis resulting in the blue and the epidermis resulting in the black. There was at least moderate agreement between pathologists for any lesion surface with less than 10% blue and black areas being significantly pigmented NM, the authors wrote. Moreover, Pizzichetta et al., related to ulceration and homogeneous disorganized patterns, homogeneous blue pigmented structureless areas, three or more colors, a mix of polymorphous vessels, and milky-red globules, as well as symmetrical shapes, were some other features significantly associated with NM 91.

### Screening and diagnosis of SSM

#### Etiology

The etiology of malignant melanoma is mainly associated with exposure to UV light, which can be regarded as a primary risk factor, especially in the presence of the susceptibility of phenotype. Furthermore, the risk of melanoma rising due to aging can be attributed to exposure to other environmental agents than only UV light. In the pathogenesis of melanoma, break periods between the initiation of exposure to the environment and the appearance of the tumor, along with many more variables, have interfered 92.

#### Epidemiology

SSM is the most common type of melanoma and accounts for approximately 70% of the total incidence of melanoma in the world 93. This melanoma originates because of the malignant transformation of melanocytes which are cells synthesizing the photoprotective melanin pigment 94. The development of a precancerous lesion is usually observed to occur gradually over several years, before a more rapid transformation in the preceding months until diagnosis. Though SSM commonly occurs on the back in men and on the legs in women, it can occur on any part of the body 95. Studies report that BRAF-mutant melanomas are more common in younger patients and mainly refer to the SSM subtype, the trunk region, and patterns of intermittent ultraviolet sun exposure 96-98. The incidence of melanoma has significantly increased over the years on a worldwide basis. In 2019, it is estimated that there were 57,220 new cases of melanoma in males and 39,260 in females in the United States, which accounts for 5.5% of the total cancer incidence and was responsible for 7,230 deaths, or 1.2% of all cancer-related deaths. The average annual number of new cases is around 132,000. According to the 2019 report from The American Cancer Society, it is estimated that 192,310 patients were diagnosed with melanoma in the U.S 99.

#### Pathophysiology

In SSM, large numbers of single melanocytes are situated in the epidermis. These melanocytes may be arranged in clusters along the dermal-epidermal junction and frequently are present in the midportion and upper layers of the epidermis as well 100. BRAF and NRAS gene mutations occur at an early point in melanoma pathogenesis and are consistently sustained during further tumor progression, taking part in the pathogenesis of invasive melanoma but also in cooperation with other mutations 101. Several studies confirmed that immune evasion mechanisms-antigen downregulation and resistance to immune cell attacks- play an important role in the uncontrollable growth of melanoma cells 102.

#### Dermoscopy

Dermoscopy is an early diagnostic technique that is non-invasive and complements the accuracy 103. One review reported that a clinical examination with the addition of dermoscopy reaches 90% specificity-95% CI: 57-98% and 90% sensitivity-95% CI: 80-95%, and went on to show just how much dermoscopy increases the accuracy of the clinical examination in identifying primary melanoma 104. Recently, dermoscopy benefitted from the technical evolution of imaging and digital cameras. The use of these new technologies allowed the creation of the so-called video-dermoscopy, paving the way for the application of this diagnostic technique for telemedicine approaches, simplifying the sharing of clinical images, and facilitating follow-up of unclear lesions 105. This comprehensive review is progress in current applications for patients, primary care providers, dermatologists, and dermatopathologists. The authors discuss various applications of image and molecular processing applied in skin cancer and point out the potential to apply AI in the self-screening of patients and improving diagnostic accuracy in non-dermatologists 106.

### Treatments

#### Surgery

Surgical treatment remains the gold standard in melanoma management. It consists in the complete excision of the scar after an excisional biopsy performed at the diagnostic stage - micro staging I - with a sufficiently large margin of healthy tissue, depending on the depth of infiltration of the lesion 107. Other lymph nodes are removed if melanoma cells are detected in the lymph nodes. Surgery is another better therapy for metastatic melanoma, but it is a supportive therapy and is often used in conjunction with other treatments as a primary treatment 108.

#### Chemotherapy

Chemotherapy was the initial treatment approach for advanced melanoma. Various chemotherapy combinations have been studied to improve the clinical responses, but no significant improvement in overall survival (OS) was detected 109. Apoptosis resistance is thought to be the main reason melanoma does not respond to treatment and while it remains one of the main treatments used in hospitals for patients with advanced, refractory, or recurrent melanoma, other treatments have replaced many chemotherapy regimens 110, 111.

#### Targeted therapy

Approximately 70% of patients with cutaneous melanoma have genetic mutations in key genes that control cell growth and cancer progression. These oncogenic mutations may be associated with melanoma cell proliferation and a malignant phenotype 109. The targeted therapy approach uses use small molecule drugs or antibodies to disrupt the mutant protein and thereby reduce the severity of the disease 112.

#### Immunotherapy

Melanoma is a cancer that generally tends to respond relatively to immune modulation 113. Various factors have been identified to explain melanoma cell sensitivity to activation by the immune system. These factors include increased tumor mutational burden caused by UV light exposure, the generation of cancer-testis antigens, and mimicry of melanocyte lineage proteins that pathogen-associated antigens 114.

## Machine Learning Applications in Skin Cancer Detection

### Acral lentiginous melanoma (ALM)

In the study proposed by Abbas et al., 115 a seven-layered DCNN model was developed using a dataset of 724 dermoscopic images collected from the Hospital at Yonsei University Health Organization in South Korea. This experiment compared and analyzed the results of the three DL models. The proposed seven-layered DCNN model achieved an accuracy of over 90%. Additionally, they applied transfer learning models like Resnet18 and AlexNet for comparison, which achieved nearly 97% accuracy. This research shows that DL models diagnose the early stage of melanoma. In this study, Islam et al., 116 proposed a CNN model that uses image preprocessing techniques, using the HAM10000 dataset, for this experiment consisting of 10,015 image files of different skin growths. The results of the study were compared with other existing models like AlexNet, ResNet, Inception V4, and VGG-16. The proposed CNN model achieved a 90.93% accuracy in training and 96.93% accuracy in testing respectively. This model works better at classification compared to other models.

Barros et al., 117 focus on supervised and self-supervised models, using datasets from ISIC data archive, Atlases, PAD-UFES-2, and Derm7pt. The models are BYOL, SwAV, MoCo, InfoMin, supervised, and SimCLR. In the DDI dataset, all models show poor results, the supervised model achieved low results, and MoCo achieved high results with 55.8% accuracy and a 12.5% f1 score. The Fitzpatrick 17k dataset shows good results, the supervised model achieved the best accuracy of 63.4% and f1-score of 41.8%. Each of the supervised and self-supervised learning approaches performs similarly on the dataset. In the PAD-UFES-20 dataset, both model types performed similarly with the BYOL model achieving a high accuracy of 59.2% and the f1 score of 27.5% respectively.

**Figure 9:**
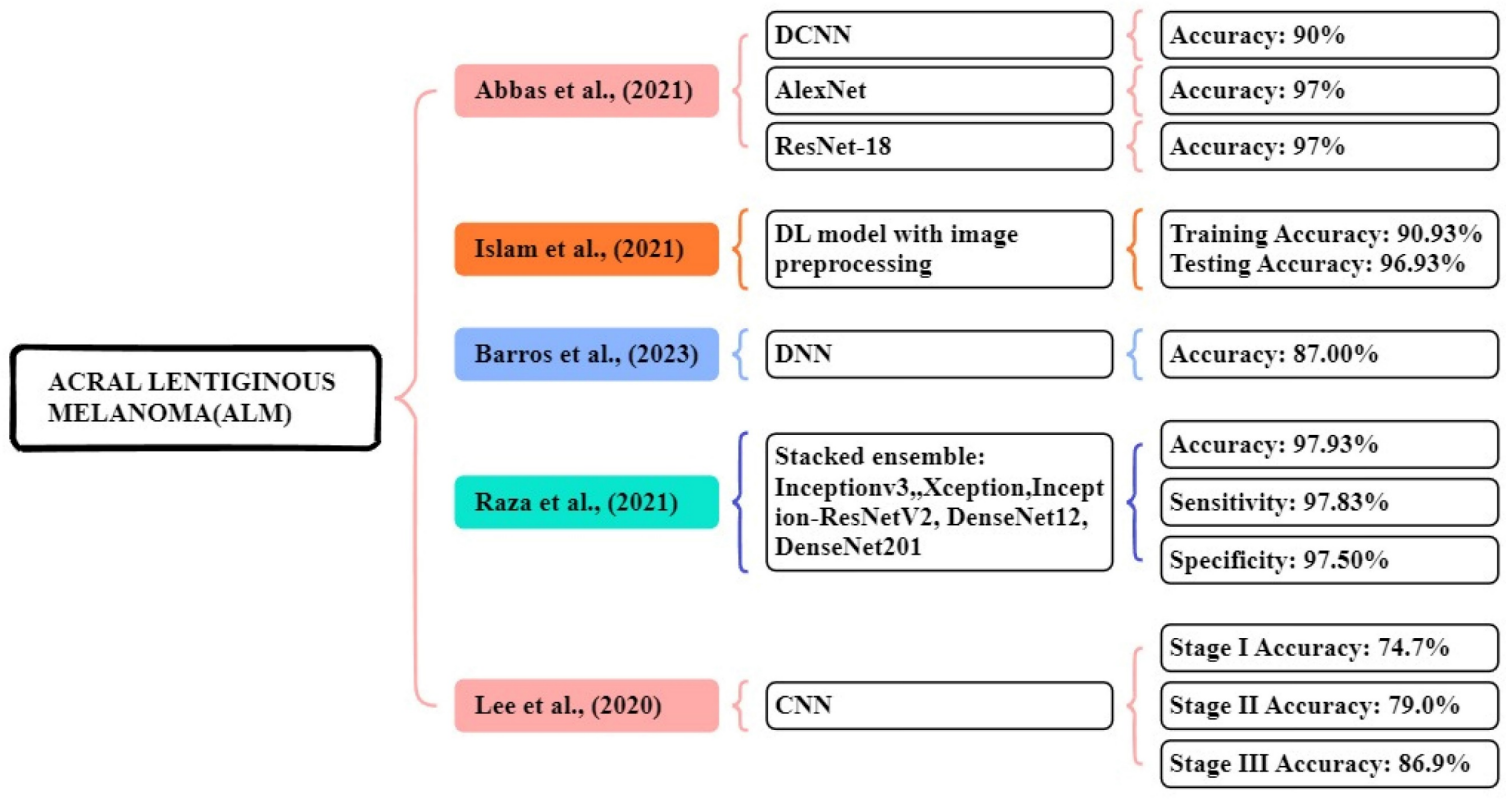
Machine Learning Overview of ALM.

Raza al., 118 focus dataset containing 724 dermoscopy images collected from Dongsan Clinic at KeiMyung University, Korea, and Severance Medical Clinic, at Yonsei University, Korea. They used stacked ensemble methods Inceptionv3, Xception, InceptionResNet-V2, DenseNet201, and DenseNet121 these models perform high accuracy of imageNet. The proposed stacking ensemble of the optimized models achieved an accuracy, sensitivity, and specificity of 97.93%, 97.83%, and 97.50% respectively. In a study focused by Lee et al., 119 used a trained dataset of 1072 dermoscopic image acral benign nevi. The system has three stages: stage I based on dermoscopic images, stage II clinical information, and stage III evaluation and probability estimated by CNN. The CNN achieved an accuracy of detecting ALM stage I-74.7%, stage II-79.0 %, and stage III-86.9% respectively. Figure 9 illustrates the methods for the early and accurate diagnosis of the ALM in these studies.

### Melanoma *in situ* (MIS)

Patil et al., 120 focus on two techniques CNN and multilayer perceptron (MLP). Using a dataset retrieved from https://dermnetnz.org/. The models are CNN-MLP architecture for multi-class classification. Their model achieved accuracy, recall, f1-score, and precision of 95.12%, 94.73%, 95.37%, and 96.05% respectively. Hussein et al., 121 implemented a CNN method for classifying melanoma skin cancer. The publicly available datasets of skin lesion images consist of 1,800 images of two types of moles. The proposed CNN model delivered a 99.99% accuracy, 99.9% precision, 99.9% Recall, and 99.99% F1 score respectively. Javaid et al., 122 focus on SVM, random forest, and quadratic discriminant for classification, using the publicly available ISIC-ISBI 2016 collection of skin images as a dataset. The SVM, random forest, and quadratic discriminant system achieved an accuracy of 88.17%, 90.84%, and 93.89%. The random forest classifier achieved high accuracy compared to other classifiers.

**Figure 10:**
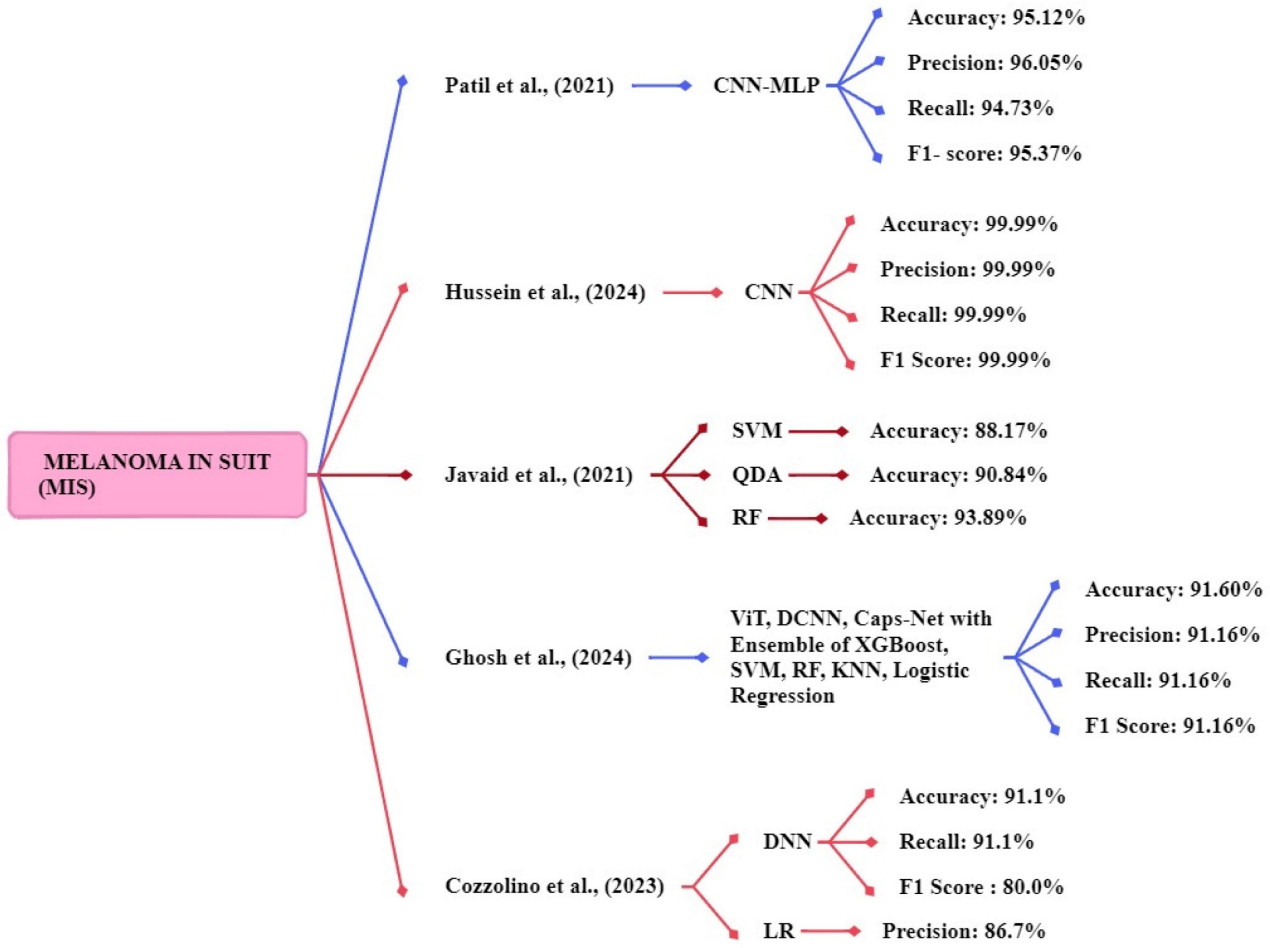
Machine Learning Overview of MIS.

Ghosh et al., 123 proposed an ensemble model that used a dataset consisting of evaluation images of 1000 and training images of 9600. The study uses ViT, DCNN, and Caps-Net to extract features from the skin image and ensemble model with five ML methods XGBoost, SVM, RF, KNN, and logistic regression. The proposed ensemble method achieved an accuracy, f1 score, precision, and recall of 91.6%, 91.16%, 91.16%, and 91.16% respectively. Cozzolino et al., 124 developed models such as LR, SVM, RF, gradient boosting (GB), kNN, and DNN. The DNN models achieved the best accuracy of 91.1%, a recall rate of 91.1%, and an f1-score of 80.0%. The logistic regression model achieved the best precision of 86.7% respectively. Figure 10 shows the methods for the early and accurate diagnosis of the MIS in these studies.

### Nodular melanoma (NM)

The study performed by Safdar et al., 125 indicated that ensemble models like DenseNet-201 and ResNet-50 significantly increased classification rates. The datasets of multiple skin lesions including Med-Node, DermIs, and PH2 have been collected for the identification and classification of lesions. The database comprises a total of 2301 images consisting of 1611 training images and 690 testing images. The ensemble models achieved an accuracy of 95.20%, ROC- AUO of 98.50%, sensitivity of 92.80%, and specificity of 96.70% in multiple dermoscopy image datasets. Winkler et al., 126 the study focus on a CNN to detect the different melanoma subtypes. Six dermoscopic image sets are used for the classifications. The CNN showed a high-level performance for NM achieving a sensitivity of over 93.3%, ROC-AUO of 92.60%, and specificity above 65% respectively.

**Figure 11:**
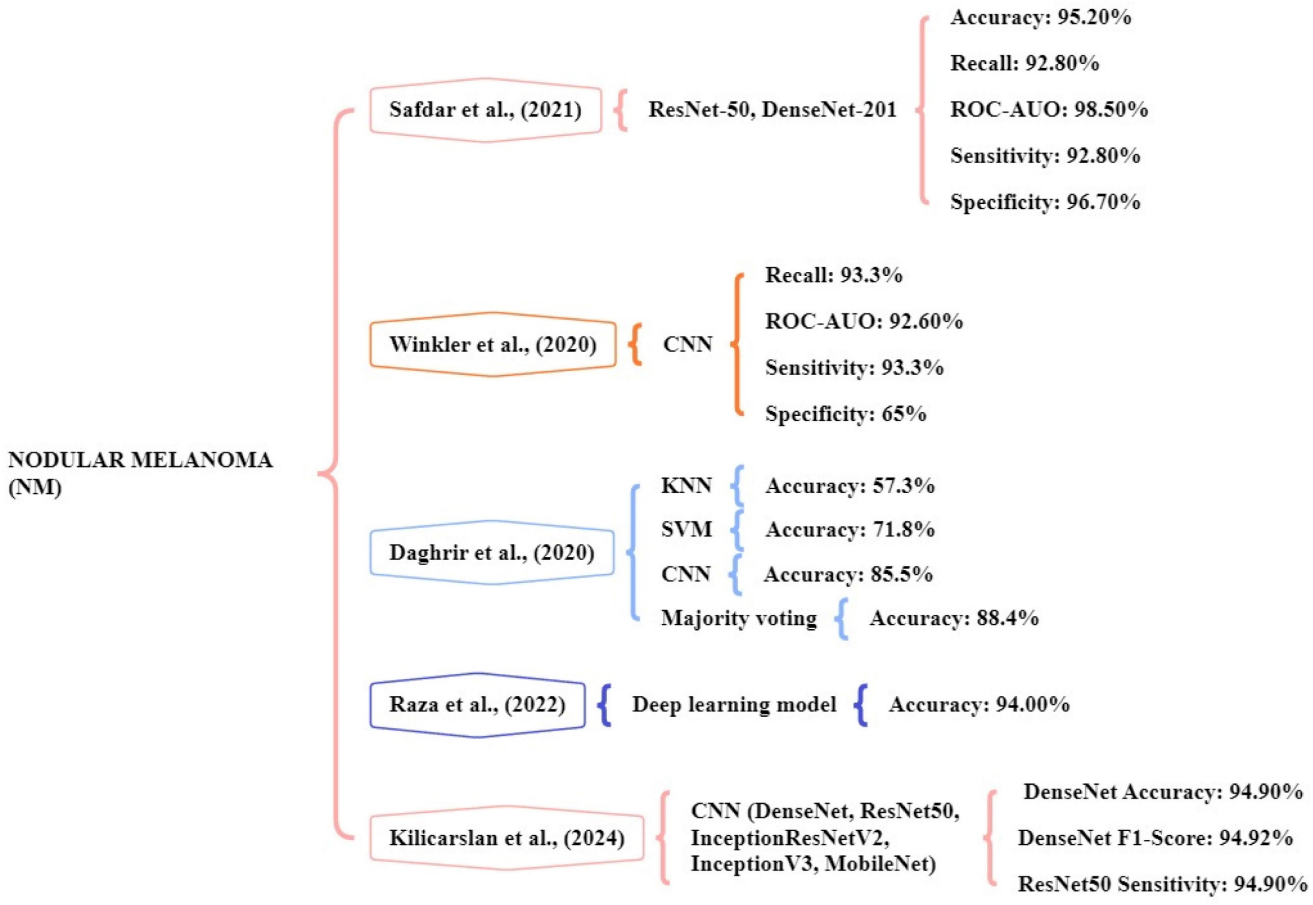
Machine learning overview of NM.

Daghrir et al., 127 focuses on different models namely KNN, SVM, CNN, and majority voting. The system utilized a public dataset of the ISIC archive, which contains 23000 images of melanoma. The system achieved an accuracy kNN of 57.3%, SVM of 71.8%, CNN of 85.5%, and majority voting of 88.4% were obtained. The majority voting methods will achieve high accuracy in detecting melanoma. Raza et al., 128 experimented with the CNN model by using the dataset of 17,805 training images using the DL model. Their CNN model achieved an accuracy of 94% for the melanoma skin cancer classification task. Kilicarslan et al., 129 the study tested five DL models Densenet, ResNet50, InceptionResNetV2, InceptionV3, and MobileNet with seven optimizers. The melanoma skin cancer dataset is used and it consists of RGB images 10,605, benign 5500, and malignant 5105. The DenseNet-SGD optimizer model delivered the best accuracy of 94.90%, f-score of 94.92%, and sensitivity of 94.03%. Figure 11 represents the methods for the early and accurate diagnosis of NM in these studies.

### Superficial spreading melanoma (SSM)

Thiyaneswaran et al., 130 proposed models consisting of three different approaches feed-forward back propagation neural network, fuzzy logic, and SVM. The PH2 and ISIC database images are used for this analysis. The images of the ISIC datasets were compared with those of existing processes, including Inception-V3, ResNet50, Inception ResNet V2, and DenseNet-201. This study achieved an accuracy of the models like fuzzy, SVM, and FFBPNN of 78%, 83%, and 90% respectively. The FFBPNN model achieved the highest accuracy for melanoma classification. Pillay et al. 131 the study focus on transfer learning by testing 14 pre-trained models to classification and diagnosis of skin cancer. The datasets used are MED-NODE, DermIS, and DermQuest to determine the model performance and this experiment consists of 376 macroscopic images. The squeezenet1-1 method achieved a high-performance accuracy of 93.42%, a sensitivity of 92.11%, and a specificity of 94.74% respectively. Kaur et al., 132 this study focus on an automated melanoma classifier using an LCNet model. The dermoscopic image datasets ISIC 2017, ISIC 2016, and ISIC 2020 were used for this study and the ISIC 2020 dataset achieved high results. The proposed LCNet model achieved an accuracy, f1-score, precision, and recall of 90.42%, 90.42%, 90.39%, and 90.41% for ISIC 2020, respectively.

**Figure 12:**
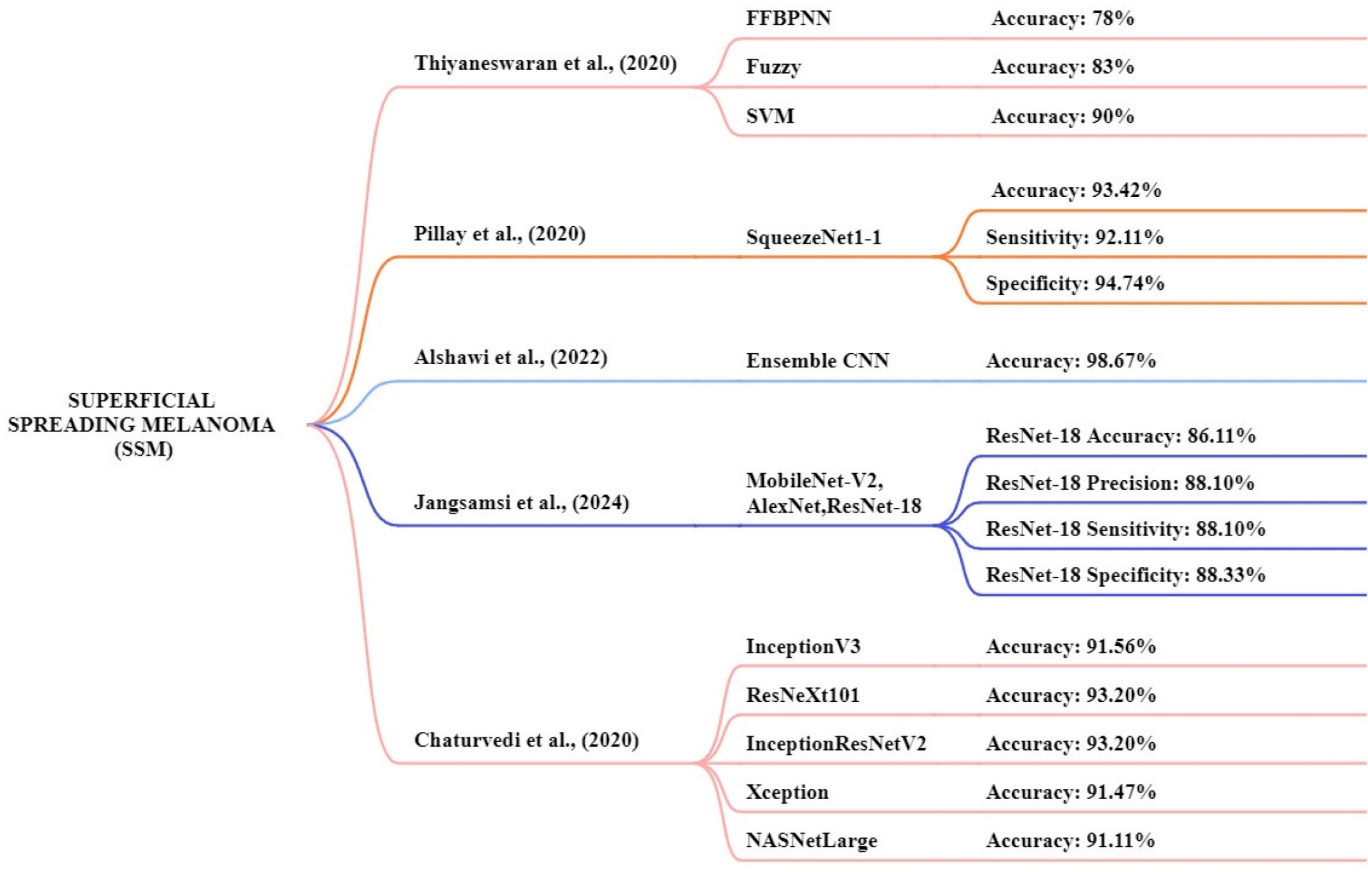
Machine learning overview of SSM.

Jangsamsi et al., 133 this research focuses on comparing three DL models AlexNet, ResNet-18, and MobileNet-V2 using a comprising dataset from the MED-NODE dermatology database. The dataset consists of 358 images of skin cancer. The ResNet-18 model achieved high accuracy, precision, sensitivity, and specificity at 86.11%, 88.10%, 88.10%, and 88.33% respectively. Chaturvedi1 et al., 134 focus CNN models like InceptionV3, ResNeXt101, InceptionResNetV2, Xception, and NASNetLarge. In this study, they utilized a HAM10000 dataset. The dataset includes 10,015 dermoscopic images of various skin cancer types. The models achieved an accuracy of 91.56%, 93.20%, 93.20%, 91.47%, and 91.11% respectively. The high-performance accuracy is ResNetXt101 and InceptionResNetV2. Figure 12 presented the techniques for the early and accurate diagnosis of SSM in these studies.

Despite significant progress in AI-based melanoma classification, some limitations remain in the analyzed models. A primary limitation is the relatively small size and uneven attributes of most training datasets, which may hinder model robustness and increase the risk of overfitting. Furthermore, most models have been developed and validated using data from limited demographic and ethnic populations, which raises concerns regarding their generalizability and effectiveness in diverse clinical settings. Variations in imaging devices, acquisition techniques, and data fidelity intensify the difficulties of model transferability. To address these constraints, it is imperative to collect larger, more diverse datasets and establish consistent review protocols to ensure that AI technologies are trustworthy, equitable, and usable for all populations. Notwithstanding the encouraging progress in AI-based melanoma detection and diagnosis, some potential obstacles persist prior to achieving broad clinical implementation. Regulatory approval processes must guarantee the safety, efficacy, and resilience of AI models across varied populations and clinical environments. Ethical considerations around data privacy, algorithmic bias, and informed consent necessitate meticulous attention to uphold patient trust and ensure equitable healthcare delivery. Furthermore, thorough clinical validation via prospective trials and real-world investigations is crucial to establish the generalizability and dependability of AI solutions. Confronting these obstacles will be essential to properly leveraging the capabilities of AI technologies in dermatology and eventually enhancing patient outcomes. A crucial issue affecting the clinical adoption of AI tools is the capacity to comprehend model decisions. Methods like Gradient-weighted Class Activation Mapping (Grad-CAM) and saliency maps offer visual elucidations of deep learning model predictions by emphasizing image areas that significantly impact classification results. These interpretability methodologies boost clinician trust through transparency, facilitate validation and error analysis, and eventually support the safer and more successful integration of AI in melanoma diagnosis.

## Discussion

### Newly emerging technologies for the early detection of skin cancer

Hyperspectral and multispectral imaging methods use optical equipment to measure several wavelength bands that are not found in the visible spectrum 135. Light absorption and reflectance properties of different parts of the skin allow these imaging methods to give a detailed overview of both structural and chemical properties of the tissues. Using this enhanced data allows for better separation of malignant from benign lesions than was previously possible with typical imaging methods. Hyperspectral and multispectral imaging improve the way lesions are examined and melanoma is detected, as they can find small skintone and shape changes in a clinical setting 136. Similarly, biosensors and molecular diagnostic systems are advanced ways to find early signs of skin cancer by looking at certain melanoma biomarkers 137. They combine biomolecules such as antibodies, nucleic acids or aptamers, to detect cancerous DNA, proteins or microRNAs in bodily fluids. When biosensors are combined with microfluidics and nanotech, this greatly increases the speed, sensitivity and available locations for diagnostic tests and may enhance traditional histopathology. Monitoring melanoma progress and therapy effects in real time, thanks to molecular diagnostics, makes it possible to personalize care for each patient. A liquid biopsy is now often used to detect and track melanoma by studying circulating tumor cells (CTCs), circulating tumor DNA (ctDNA) and extracellular vesicles found in the blood or in various bodily fluids 138. This approach allows doctors to analyze the tumor's molecular features without taking out pieces of the tumor. With liquid biopsy, metastasis can be detected early, the variability of tumors can be studied and how therapy is working can be monitored in real time 139. Manufacturers have greatly improved the accuracy of liquid biopsy for melanoma thanks to new technology. Nanotechnology-enhanced imaging methods, in conjunction with optical coherence tomography (OCT), provide high-resolution, depth-resolved viewing of skin microstructures, hence enhancing the identification of early melanoma lesions 140. Nanoparticles can be designed as contrast agents or molecular probes to selectively target certain tumor markers, hence improving OCT signal contrast and specificity. OCT delivers cross-sectional images of the skin with micrometer-level resolution, facilitating noninvasive evaluation of lesion depth and morphology in real time 141. The integration of nanotechnology with OCT presents significant potential for enhancing traditional imaging techniques and enabling more precise, early-stage melanoma detection.

### Established and emerging methods for differentiating benign and malignant lesions

Diagnosing benign from malignant skin lesions is easier because of these types of diagnostics which rely on analysis of specific genomic, proteomic and metabolomic profiles 142. Experts use genetic markers and especially changes in BRAF and NRAS genes, to learn more about how melanoma develops and progresses. In proteomic studies, characteristic changes in proteins are observed in cancer cells, while metabolomic analysis finds differing metabolic ways in cancer. The use of biomarkers provides more clarity about lesions than just their appearance, helping with early and correct diagnosis. By using high-throughput sequencing and mass spectrometry technology, molecular diagnostics can become more accurate and help with choosing personal approaches to care and treatment. RCM, MPM and hyperspectral imaging allow advanced visualization of the skin's structure and cellular shapes without invading the skin. RCM lets experts see inside the skin in real time and at nearly the same quality as a biopsy, helping to identify cancerous cells without a biopsy. With endogenous fluorophores, MPM captures images that highlight the energy use and organization of tissues. Hyperspectral imaging gives a wide range of frequencies, so it can detect the biochemical differences between tumors and normal tissues that help tell them apart. They add to the reliability of diagnosis and obviate unnecessary invasive treatments, accompanying traditional medical and dermoscopic evaluations. Combining the findings of a clinical exam, images and molecular information often results in more accurate diagnosis of skin lesions and reduces incorrect positive or negative test results. Employing multimodal data fusion, we are able to merge morphological, functional and molecular information to assess the lesion status carefully 143. The use of machine learning and similar algorithms supports the efficient processing of different types of data which helps build dependable diagnostic models. When an integrated approach is used, doctors can create unique treatment plans that benefit each patient's care.

### Newly emerging therapeutics for the treatment and prevention of skin cancer

Concentrating on important systems in the body that fuel tumor growth and life has brought major changes to melanoma treatment. Using BRAF and MEK inhibitors which are small molecule inhibitors, helps to stop diseases related to melanoma in patients with these gene abnormalities 144. On top of that, monoclonal antibodies designed to interfere with signaling or attack antigens on cancer cells are highly effective and cause less harm to the body. As a result of using targeted strategies, melanoma patients have improved reaction to treatment and live longer than those who use conventional chemotherapy. Immunotherapy is now often given in melanoma treatment, relying on the patient's immune system to seek out and kill cancer. Anti-CTLA-4 and anti-PD-1 antibodies are immune checkpoint inhibitors that boost immune cells and encourage them to fight cancer 145. Cancer vaccines and therapies such as transferring tumor-infiltrating lymphocytes are meant to enhance how the immune system recognizes cancer cells. Numerous patients now benefit from a longer and more successful treatment due to the standard-setting value of these medications in advanced melanoma. It uses light therapies called photodynamic therapy and lasers to treat selected cancerous cells without harming healthy tissues nearby. The approach involves using drugs that build up in cancer cells which are then exposed to light of the right color to make toxic oxygen species. Laser therapy uses direct and focused light power to carefully remove malignant tumors 146. Especially with early or outward skin cancers, these methods are useful as either main or complementary treatments to surgery. Skincare precautions are designed to decrease the risk of skin cancer with prevention drugs, better habits and new vaccines. Nicotinamide and retinoids are drugs that have shown they help decrease the risk of non-melanoma skin cancers. Public health programs underscore sun protection practices, such as the application of sunscreen and the avoidance of UV exposure, as essential strategies for melanoma prevention. Furthermore, vaccination research is investigating preventative and therapeutic strategies aimed at melanoma-associated antigens to elicit enduring immune protection. These methods combined form a comprehensive strategy for skin cancer prevention.

## Conclusion

Melanoma includes subtypes like ALM, MIS, NM, and SSM which present significant challenges for early detection and accurate diagnosis. The systematic review paper has considered various machine-learning techniques for detecting and classifying skin cancer. All those techniques are noninvasive. Recent advancements in deep learning, particularly CNN are highly effective in improving diagnostic accuracy. By integrating advanced imaging techniques with dermoscopic, healthcare providers can diagnose melanoma earlier and improve accuracy, thereby providing instant treatment and better patient care. This technique has greatly improved the accuracy of skin cancer detection, the diagnostic process, and clinicians diagnosing high-risk lesions. There is strong potential for further development, particularly in enhancing AI models by integrating a broader range of multimodal input data, including genetic markers, patient histories, and advanced imaging techniques such as hyperspectral and multispectral imaging. Challenges will survive, especially in addressing class imbalance when the dataset expands to include various skin types and melanoma subtypes. The integration of AI tools into telemedicine presents a valuable opportunity to increase early screening, especially in rural areas. The effective incorporation of AI-driven melanoma detection tools into clinical practice will mostly rely on thorough prospective validation trials carried out in actual healthcare environments. These studies are crucial for thoroughly evaluating model performance, safety, and usefulness among varied patient populations and clinical workflows. Furthermore, the continual enhancement of AI algorithms via persistent learning and feedback systems will be essential to uphold their precision and pertinence. Subsequent research ought to investigate multi-institutional cooperation to promote data sharing and standardization, thus improving model robustness and generalizability. These initiatives, alongside a focus on ethical, regulatory, and practical factors, will facilitate AI's integration as a crucial element in tailored melanoma management. In conclusion, the research in AI, advanced imaging technologies, and telemedicine provides strong improvements in melanoma detection and diagnosis. The advancement is considered to improve early skin cancer detection and provide more personalized treatment options, improve patient outcomes and further advance the management of skin cancer.
